# Efficient Forest Fire Detection Index for Application in Unmanned Aerial Systems (UASs)

**DOI:** 10.3390/s16060893

**Published:** 2016-06-16

**Authors:** Henry Cruz, Martina Eckert, Juan Meneses, José-Fernán Martínez

**Affiliations:** Research Center on Software Technologies and Multimedia Systems for Sustainability (CITSEM), Universidad Politécnica de Madrid, Alan Turing St., Madrid 28031, Spain; martina.eckert@upm.es (M.E.); juan.meneses@upm.es (J.M.); jf.martinez@upm.es (J.-F.M.)

**Keywords:** forest fire detection, color index, mobile surveillance, UAS, UAV, drone, real-time, cost-efficient

## Abstract

This article proposes a novel method for detecting forest fires, through the use of a new color index, called the Forest Fire Detection Index (FFDI), developed by the authors. The index is based on methods for vegetation classification and has been adapted to detect the tonalities of flames and smoke; the latter could be included adaptively into the Regions of Interest (RoIs) with the help of a variable factor. Multiple tests have been performed upon database imagery and present promising results: a detection precision of 96.82% has been achieved for image sizes of 960 × 540 pixels at a processing time of 0.0447 seconds. This achievement would lead to a performance of 22 f/s, for smaller images, while up to 54 f/s could be reached by maintaining a similar detection precision. Additional tests have been performed on fires in their early stages, achieving a precision rate of *p* = 96.62%. The method could be used in real-time in Unmanned Aerial Systems (UASs), with the aim of monitoring a wider area than through fixed surveillance systems. Thus, it would result in more cost-effective outcomes than conventional systems implemented in helicopters or satellites. UASs could also reach inaccessible locations without jeopardizing people’s safety. On-going work includes implementation into a commercially available drone.

## 1. Introduction

Current environmental conditions have recently been producing more frequent and severe wildfires, causing the destruction of sizeable forested areas every year. According to [1], in 2013, in the Sierra Nevada (USA) alone, 104,131 ha of forest were burned down, and the European Forest Fire Information System (EFFIS) [2] reports at least 176,116 ha of destroyed forests in Europe, the Middle East, and North Africa in 2014. There have also been studies proving that the fires have contributed to the rise of global temperatures and climate change [3,4]. They also indicate that fires will occur even more frequently over the next few decades [2,3,4,5]. As a consequence, there will be an incremental loss of human life, as well as damage to the economy and the environment, e.g., in biodiversity. Therefore, forest surveillance and control is an increasingly important issue, and the challenge is to find an efficient way in which to reduce forest fires. Various techniques are already in place, such as those employing active and passive sensors, which create alerts when fires occur: e.g., ionization smoke sensors, radio acoustic sensors, projected (optical) beam smoke sensors, flame detectors (for ultraviolet, infrared or visible spectrum) and others [6,7,8,9]. These sensors obtain a variety of signals, which are products of the combustion process.

### 1.1. Video Surveillance for Forest Fire Detection

The analysis of video information is an interesting and very promising option for carrying out surveillance tasks real-time. Normally installed on towers, conventional cameras can provide a continuous sequence of video frames of an alerted area. Those systems are composed of four parts: the optical sensor, a computer for image processing, the image processing software, and a communication network to transmit information [7].

To the best of our knowledge, one of the first works on fire detection based on image processing was presented by [6]. Here, a thresholding process distinguished fire from non-fire in select Regions of Interest (RoIs) to save computational costs. As this was one of the first solutions, it presented limitations in detection due to confusions in classification. Furthermore, it was designed for fixed environments. Since then, multiple proposals have been developed considering the following characteristics (amongst others): intensity changes, Gaussian combinations, background subtraction or automatic classification systems [10,11,12,13]. The techniques have been worked out both for images representing the visible spectrum as well as for those obtained in near infrared (NIR).

The work presented here focuses on the detection of RoIs, which in this case are flames and smoke, based on the calculation of color indices. Therefore, the images obtained in the Red, Green and Blue (RGB) three-band space, are transformed into a grey-scale band, by a process of arithmetic operations between image components, which enhance the desired colors. This result is binarized by a thresholding process, separating the region of interest (RoI) from the rest. Color indices are frequently obtained as Vegetation Indices (VI) and are applied in machine vision tasks for agricultural automatization, especially for detections of crops and weeds [14,15].

The reason for using these methods is due to the relationship between the capacity of effective detection and minimum execution times. They permit real-time detection and evaluation and therefore the possibility of using additional data obtained from the images, for instance, the size of the fire, coordinates, extension velocity, *etc.*, which are useful and essential for rapid reaction. The data could be represented easily as augmented reality information on mobile platforms like tablets or mobile phones, so that help could be called from the nearest place and helpers could communicate and coordinate better, by relying on identical and instantly updated information.

### 1.2. Aerial Forest Fire Surveillance

One of the main problems of fixed surveillance systems (e.g., cameras located on towers), is their limited capacity for monitoring extensive areas. Traditionally this limitation has been tackled by employing satellite and aerial transportation systems. Drawbacks of those systems include elevated costs, as well as technological, operative and climate limitations (clouds and other atmospheric factors limit information acquisition) [16,17]. Moreover, satellite systems do not represent the best information source due to a poor spatial *vs.* temporal resolution [9,16,18]. In addition, monitoring via planes can be costly, and the probability of accidents rises in the case of fires [19].

A very promising alternative, due to much lower costs and risks, is the use of small Unmanned Aerial Systems (UASs), also called drones or Unmanned Aerial Vehicles (UAVs). UASs are becoming increasingly sophisticated and frequently offer autonomous control. Over the last few years, in particular, some research groups employed UASs in forest fire detection tasks and studies [9,16,19,20]. Therefore, optical as well as NIR cameras have been applied together with wireless networks for real-time or near real-time transmission [9,16,20,21]. Furthermore, all of those studies proposed the use of costly sensors, which leads to an unfavorable cost-benefit relationship that was even worse than for traditional systems [18].

Therefore, this study proposes the use of low-cost camera equipment and fast detection algorithms via a color index, specially designed for fire detection. In recent years, color indices have already been employed to distinguish cultivated areas from the rest. For example, in [15], the combination of color indices with Hough transform achieves a detection rate of 89.3% with average processing times of 4.678 ms for image sizes of 1392 × 1044 pixels and also [22,23] have reported efficient vegetation detection. Although the relation between spatial and temporal resolution will depend on each flight condition, those capacities and advantages of using color indices permit their use in UASs for forest fire detection. Furthermore, the boom in drones, for usage in professional and private sectors, cut their prices and those of related equipment continuously, so that more and more professional equipment is now available at vastly reduced prices. Moreover, due to their reduced size, these vehicles are very flexible and can reach difficult locations better than standard planes [9]. A UAS, for instance, could get quite close to a fire during its early stages, thereby sending an alarm and geo-positional information, including high-resolution imagery with 180° vision.

Nevertheless, there remain challenges to achieve this [24], e.g., one of the newest proposals was found not to perform in real-time.

### 1.3. Motivation for this Work

All over the world, many institutions involved in environmental protection and surveillance, citizen’s security or investigation, require fast and efficient methods to detect forest fires. In particular, there is a certain demand for surveillance of small and medium territories, affordability, as well as real-time responses (alarms) [1,2,9]. A way to meet those expenses is through the implementation of efficient and effective tools that can be used on mobile platforms such as UAVs/UASs. As such, a key motivation for this work has been to smooth the way for efficient hardware and software solutions that could be applied to forest monitoring and control and, above all, could enable relevant parties to react quickly and appropriately to a forest fire event. As a consequence, the two most important contributions of this work are:

1. The development of two new color indices, the Fire Detection Index (FDI) and Forest Fire Detection Index (FFDI), which allow for the detection of flames and smoke;
2. The achievement of efficient processing times to facilitate real-time implementation on a mobile platform.

The rest of the article is organized as follows: Section 2 explains the process to generate the color indices FDI and FFDI for the detection of flames and smoke. Section 3 provides an overview of the complete detection process. In Section 4, the results of experiments in different environments are presented, evaluating multiple metrics for detection precision and processing times. In Section 5, a brief discussion is added. Finally, Section 6 concludes the work and outlines directions for future work.

## 2. Generation of the Fire Detection Indices

This chapter describes the steps required to create the indices mentioned above: FDI as a general Fire Detection Index and FFDI as a more specialized index for detecting forest fires, *i.e.*, in the environment of green colors. First, to understand the general idea of color indices, some background information is given about the classical Vegetation Index (VI), which is used to identify Excess Green colors (ExG). This index is also later integrated into FFDI.

### 2.1. Background Information

Multiple types of color indices have been proposed during the last two decades, mainly as VIs to identify specific plants, crops or weeds in agriculture remote sensing tasks. The indices, in fact, are grayscale images, obtained by a process of simple arithmetic operations with the three color components RGB, which are used to enhance certain color tones while attenuating other unwanted ones. The results can then be easily segmented by thresholding, separating the region of interest from the rest.

Woebbecke et al. presented the initial work in 1995 [25], proposing five different indices for plant classifications. Amongst them, the ExG index stands out and is attractive to this study, as it enables vegetation to be extracted from the background:

(1)  ExG=2g−r−b

where *r*, *g* and *b* represent the normalized RGB components. Since this time, many other indices have been developed and efficiently employed for segmentation, with thresholding processes based on Otsu [26] as in [14,15,23,27,28,29]. This work takes advantage of those experiences and results, and proposes a similar index, but one based on a different principle, which is the enhancement of colors contained in flames and smoke while attenuating the vegetation parts.

### 2.2. Creation of Color Indices for Forest Fire Detection

The principle of the indices proposed in this work is based on that of VIs, with the difference being that, instead of green colors, the reddish tones contained in flames and smoke will be salient in the resulting grayscale image. The steps necessary to follow, during the index generation process, are described below and could be applied as a general methodology for creating other such indices, as required. The process consists of normalization and analysis of the relation between components and arithmetic operations, leading to the indices.

#### 2.2.1. Normalization

The first step is the normalization of the RGB components to achieve a better robustness against different lighting conditions. Therefore, all components are first normalized by their maximum possible value (255) and afterward by the sum of RGB, leading to the following expression:

(2) $$r=\frac{R}{R+G+B}\;,\;g=\frac{G}{R+G+B}\;,\;b=\frac{B}{R+G+B}$$

This process has repeatedly been described in multiple publications, such as [14,15,25,28].

#### 2.2.2. Relation between Color Components

To find the equations for the arithmetic operations, carried out between the RGB components to enhance the desired color tones, the most important step is analyzing the relationship between the components inside the regions of interest. This means that the color tones that compose the target group to be identified have to be analyzed regarding their composition between channels so that those with a stronger influence can be enhanced and others attenuated accordingly. For example, in Equation (1) for ExG, the green component is reinforced by duplication, while red and blue are subtracted. In this way, those colors composed of more green than red and blue appear as brighter; others, containing red and blue, appear as darker gray values in the resulting ExG image.

The problem tackled in this study is that of detecting areas of fire, which includes flames, smoke, and steam. Therefore, the color ranges to detect includes yellow, red and orange tones of the flames, as well as white and grayish parts of smoke and so forth. Covering a wider range, the analysis of these colors will not lead to a clear predominance of one channel, such as in green color extraction, but rather to a fixed relationship, which enables the establishment of an equation that enhances the desired areas.

**Figure 1:**
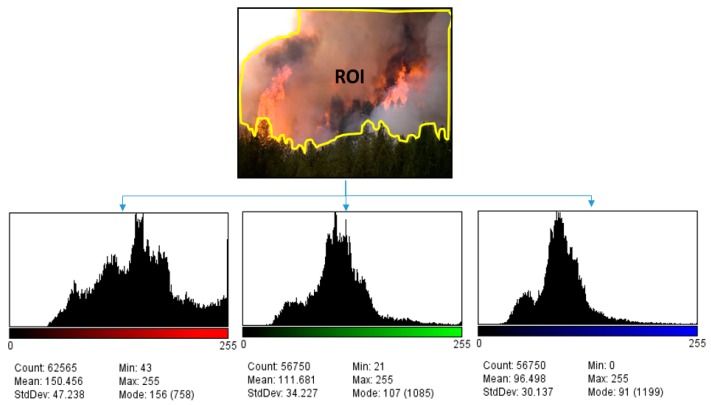
Example histograms for color distribution in areas presenting flames and smoke.

The analysis has been performed over 30 images obtained from the European Fire Database [30] and the Forestry Images Organization Database [31]. The images were selected considering aspects like image perspectives (similar to that obtained by a UAV) and containing a combustion of flame and smoke. In all 30 images, the RoI has been manually marked as shown in the example in Figure 1. In the RoI, all the pixels forming the flames and smoke area have been included. In some cases, border pixels have also been included where flames and vegetation are fusing together, to include the maximum amount of elements which could provide information.

For the selected pixels, the histogram distributions of the three components have been analyzed, calculating the average values of each. The histograms illustrate the distribution of color intensities inside the RoI for each component; here, the *x*-axis represents the value between 0 and 255 and the *y*-axis the number of pixels found for each value. Finally, all mean values have been averaged over the 30 test images with the result that *R_m_ > G_m_* and *R_m_ > B_m_*. Consequently, the general relation between the normalized components can be established as follows:

(3) r>g ∧ r>b

Due to this relationship, the equations to obtain the proposed color indices can be established, as outlined in the following sections.

#### 2.2.3. Fire Detection Index (FDI)

Equation (3) shows that the colors of interest, *i.e.*, those representing a combustion process, are composed of higher red channel values than blue and green. This knowledge leads to the conclusion that the way to achieve a gray scale image, which represents the desired colors with brighter values, would be through arithmetic operations giving more weight to the red channel than to the rest. Therefore, as a first step, the green and blue component images are subtracted from the red one: *A = r – b* and *B = r –* g. Afterwards, both results are added (*A + B*), leading to the Fire Detection Index:

(4) FDI=2r−g−b

Example images are presented in the Section 3, as parts of the whole detection process. The flame portion is represented with much lighter values than the smoke because the reddish orange color of the flame contains a much higher amount of red values than the gray-white part of the smoke.

FDI, as it is, could be employed quite well if there was a flame present in the image. Even quite small, early stage fires could be detected, as long as the environment does not contain similar colors. Nevertheless, most fires will not be observed nearby when they start, and from larger distances no flames will be visible—only the smoke. As a consequence, it is crucial to include the detection of the grey-white areas as well and to distinguish them clearly from the background. To achieve this aim for forested environments, the FDI has been extended to cope with a vegetative background, the development of which is described in the following section.

#### 2.2.4. Forest Fire Detection Index (FFDI)

A twin-track approach has been followed to achieve a wider detection range that includes the area of smoke and also achieves a real discrimination from the green tones of the background. On one hand, the region of combustion was made more salient via FDI and on the other hand, the vegetated area was attenuated, resting the ExG proposed by Woebbecke [24] and described in (1). In this way, two indices are combined, both of which are valid and able to achieve segmentations of the RoI, for which they were created: flame and smoke via FDI and vegetation via ExG. By subtraction, one of these indices, in this case, ExG, is validated negatively, which further enhances FDI against the green values.

Additionally, to achieve more flexibility and to give even more importance to the colors contained in fire and smoke, a regulative weighting factor *rho* (*ρ*) has been added. The resulting index is called Forest Fire Detection Index:

(5) FFDI=ρ×(FDI)−ExG

(6) FFDI=ρ(2r−g−b)−(2g−r−b)=r(2ρ+1)−g(ρ+2)+b(1−ρ)

**Figure 2:**
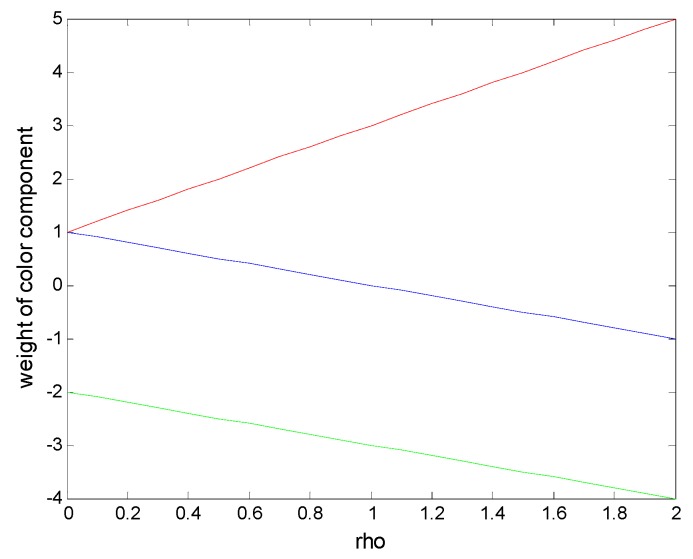
Influence of *ρ* on the weighting of each color channel in the Forest Fire Detection Index (FFDI).

Here, the parameter *ρ* works as follows (see also the illustration in Figure 2): for ρ *= 0,* the red and the blue colors are not altered, only green is subtracted two times such that: *FFDI = r − 2g + b*. If ρ *= 1*, the blue channel is completely cancelled out, while red and green are equally weighted and subtracted, such that equal or similar components would counteract each other. This leads to *FFDI = 3r − 3g*. A further increase of *ρ* causes an even higher ponderation of red, while the other two channels are weighted negatively. e.g., ρ *= 2* results in *FFDI = 5r − 4g − b*.

This means that lower values of *ρ* enhance colors with a high amount of red compared to the other channels, *i.e.*, reddish tones. Bigger values of *ρ* between 0 and 1 (0 < *ρ* ≤ 1) cause an enhancement of the yellow and brown tonalities, which are around the red ones because the blue channel is canceled out. With a further increase of *ρ*, up to two brighter colors (bright yellow in the center of the flame) and whiter or grayer ones (smoke) will be detected because they are composed of almost equal RGB values. For better distinguishing them, it is necessary to increase the red component.

Table 1 shows an analysis of some color samples extracted from the areas of flame and smoke. Here, the RGB values can be examined and compared with their normalized values. In addition, ExG and FDI have been calculated as well as FFDI for different values of *ρ*. Afterward, the threshold for binarization (see Equation (8)) which was calculated for each image and will be explained in Section 3, has been applied to show which samples would be converted into detected ones (1 = white) or not detected ones (0 = black). As can be seen, the most reddish tones are always detected, the larger the *ρ*, the more surrounding colors are “caught”, but only if the red component predominates, compare, e.g., brown gray, gray and bright gray. For *ρ* = 2, brown gray and bright gray are detected, but the “normal” gray not, as RGB are too similar. The method does not capture very light gray values or those with more blue, such that smoke will be detected only in the surroundings of the flames, but neither in the sky nor in an arousing fire that still has no flame.

**Figure 3:**
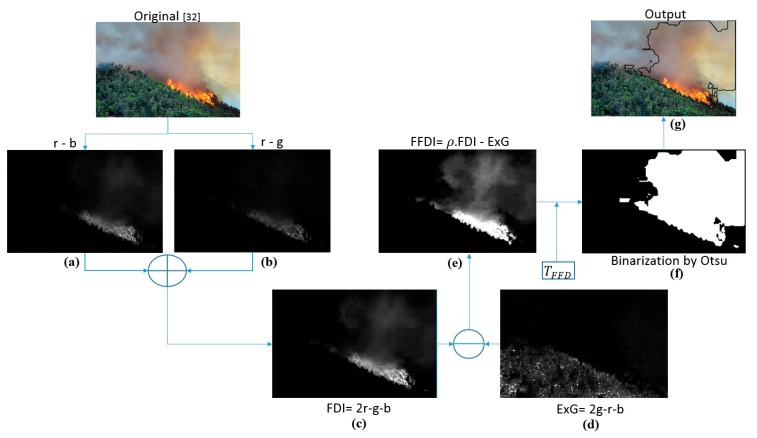
Diagram showing the forest fire detection process with color indices. Calculation of: (**a**) *r* minus *b*; (**b***) r* minus *g*; (**c**) FDI (Fire Detection Index); (**d**) ExG (Excess Green colors) and (**e**) FFDI; (**f**) binarization; (**g**) detected fire region. The original image in this figure was taken from [32].

The differences of FDI and FFDI can be observed in Figure 3. Image (e) shows the FFDI with a much more precise result than FDI in the picture (c). Image (d) shows ExG with the green areas in a wide dynamic range. The following chapter describes the complete detection process where the FFDI outcome passes through a thresholding processed to obtain a final binary segmentation.

## 3. Detection Process

The entire process of detection is composed of the following steps:

1. Image acquisition (individual images or consecutive video frames)
2. Extraction of color components R, G and B
3. Normalization of color components leading to *r, g* and *b*
4. Calculation of detection indices FDI followed by FFDI
5. Binarization through application of T_FFD_ (Forest Fire Detection Threshold)
6. Labelling of segmented regions

Examples of intermediate results obtained in this process are shown in Figure 3.

For a robust and precise detection of the RoI, the binarization of the formerly obtained index is crucial. In this step, the colors sought, which are presented in the FFDI image as elevated gray values, have to be separated from the rest (background). In many other studies, this step has been successfully resolved with help from the Otsu method [29]. Here, the same method has been employed to create the Forest Fire Detection Threshold T_FFD_ by averaging the standard deviations of FDI and ExG:

(7) $$\;T_{FFD}=\;\frac{\sigma_{FDI}+\;\sigma_{ExG}}{2}$$

After applying the FFDI to the original image, all pixels values greater than or equal to the T_FFD_ threshold are labeled with “1”(white) and the rest with “0” (black). Therefore, the final image shows the RoIs as white areas as can be seen in Figure 3f:

(8) $$g\left(x,y\right)=\;\{\begin{array}{c} 1\text{ if FFDI}\geq T_{\text{FFD}}\;\\ 0\text{ if FFDI}<T_{\text{FFD}}\; \end{array}$$

For better visualization of the results, the detected regions have been outlined with black borders in the original images, as shown in the example in Figure 3g.

## 4. Evaluation

In this chapter, different evaluative tests are presented. In the first section, the influence of factor *ρ* is analyzed according to the precision of detection of flame and smoke. In the next two sections, the algorithm’s performance is assessed over forested areas: from huge distances to test the areal perspective (UAS’s view) and from short distances to test a nearby view of initiating combustion processes. The last section describes results from images obtained in environments other than fire, to show the versatility of the algorithm.

In all tests, detection precision has been compared with processing times to determine the usefulness for real-time implementation in UASs.

The digital treatment of the images was carried out with MATLAB^®^, running on an Intel Core i5@3.1 GHz, 8 GB RAM computer (GBT Inc., City of Industry, CA, USA). For the tests, different image content, views and formats have been compared.

### 4.1. Influence of Factor ρ in the Detection of Flame and Smoke

As formerly presented in Section 2.2.4, the FFDI has been designed with the help of a factor *ρ* which regulates the strength of FDI against ExG to distinguish more or less the quantity of the smoke area, surrounding the flame, from the forested background. This factor emerged during the first trials and had been shown to be a useful complement to the algorithm for different applications or requirements.

**Figure 4:**
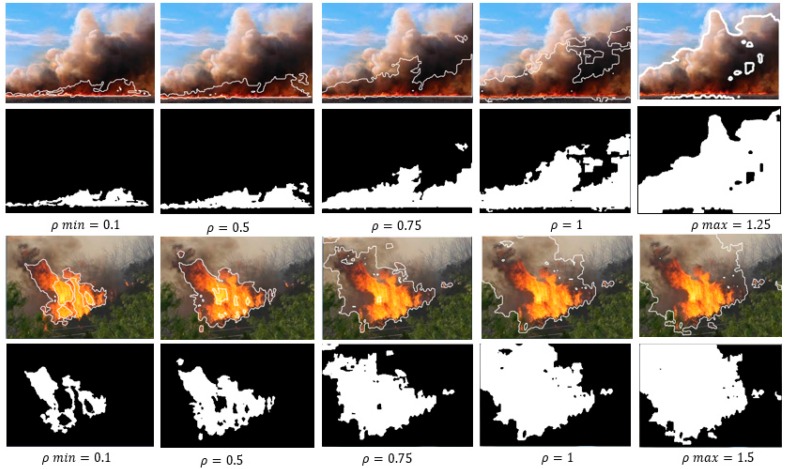
Influence of *ρ* in detection of flame and surrounding smoke.

Analyzing the effect of *ρ* in the range *0 < ρ < 1*, the following can be said: *ρ = 0* does not apply as it would mean an exclusive application of the negative ExG index. When incrementing *ρ*, a minimum value has to be reached first. It should be different for every image due to color saturations and lighting conditions to detect the flame reliably; this value is called *ρ_min_*. A further increase includes more and more white and gray colors (that contain red tonalities) because green and blue are further subtracted, which explains the increase of the detected smoke area (see Figure 4). Finally, a maximum *ρ* is reached (*ρ_max_*), which establishes the best possible detection of flame plus smoke. A further enhancement would not change the result.

Figure 4 shows how the detected area grows with an increment of *ρ* from *ρ_min_* = 0.1 (flame without smoke) to *ρ_max_ = 1.5* (flame and full area of smoke). According to Equation (6), *ρ = 1.5* results in *FFDI = 4r − 3.5g − 0.5b*, a further enhancement would put more and more weight on red values, but would not lead to differences in the segmentation result. As can be seen in the furthest right images, nearly the entire smoke area has been detected, with only a few dark areas missed and the distinction from the background is quite precise.

### 4.2. Aerial Perspective and Large Distance Evaluation

Multiple tests were undertaken on database images to evaluate the key goal of this paper using the proposed method to detect forest fires via UASs. A total of 50 images were selected from the European Fire Database [30] and the Forestry Images Organization Database [31] in jpeg format and different sizes near to but not exceeding 1920 × 1080 pixels. All are shot from various aerial perspectives and angles. Size and format would correspond to those of video frames obtained by commercially available drones (e.g., Bebop Parrot, Parrot SA, Paris, France), such that the evaluation conditions would be as close to reality as possible.

For the analysis of the performance and processing time, all images had to be resized to 1920 × 1080 pixels. Afterwards, all images were resized to: 2/3: (1280 × 720), 1/2: (960 × 540), 1/4: (480 × 270) and 1/8: (240 × 135) using the Matlab function “imresize”.

**Figure 5:**
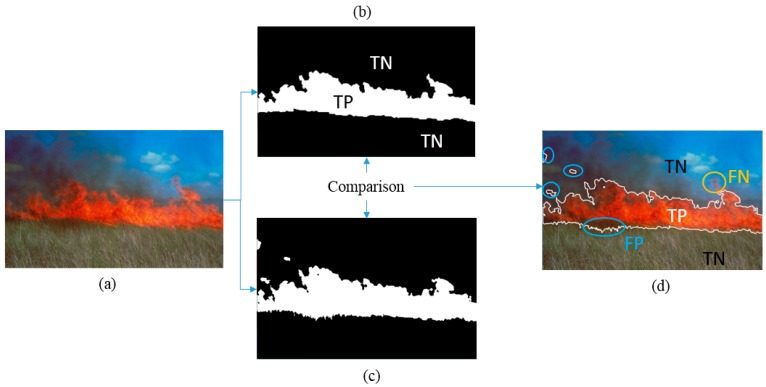
Illustration of classification of the resulting segmented regions. In (**a**) original image; (**b**) ground truth; (**c**) detection by FFDI; (**d**) comparison result.

The detection accuracy was evaluated with the help of a confusion matrix, *i.e.*, by comparing the segmentation results with a ground, truth segmentation, following the methodology of pattern comparison and precision analysis. Therefore, True Positives (TP), True Negatives (TN), False Positives (FP) and False Negatives (FN) are counted. TP are correctly detected pixels, *i.e.*, those assigned to areas containing only a flame or flame and smoke. TN are pixels correctly assigned to the rest of the image. Consequently, FP and FN are pixels which are not correctly assigned (FP = false fire detection, FN = false non-fire detection). Figure 5 shows visually how the detected areas are compared with the ground truth image classified for the statistical measures.

**Figure 6:**
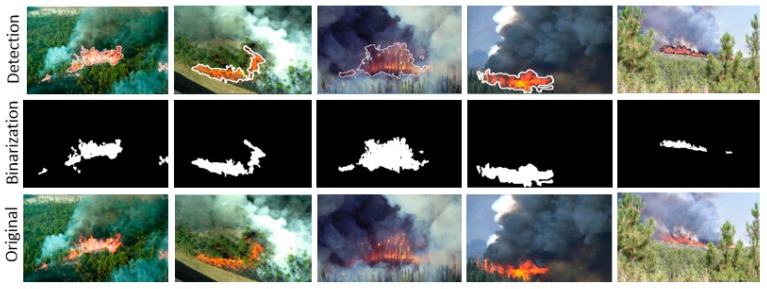
Examples for forest fire detections from aerial perspective.

The ground truth segmentations have been obtained by roughly segmenting the areas of the flame manually. Here, the smoke has not been considered, as it is too difficult to distinguish its limits and because the method works only for smoke colors with a certain amount of red, such that whiter or bluer tones cannot be detected. As a consequence, the tests have been performed only for *ρ_min_* which is aimed at detecting only the flame. In Figure 6, a selection of segmentation results is shown for the image size of 1920 × 1080 pixels and *ρ_min_* = 1.

For quality evaluation, the following five commonly used metrics based on statistical tests of TP, FP, TN and FN were calculated: Precision *p* = TP/(TP + FP), Recall *r* = TP/(TP + FN) and the Dice Index DI = 2(p·r)/(p + r), characterize the general performance of the detection, especially DI evaluates the overall detection accuracy. The Jaccard Index (JI) as follows JI = TP/(TP + FP + FN) indicates the similarity rate between two segmentation areas, and the Manhattan Index (MI) studies the similarity rate of the entire image MI = (TP + TN)/(TP + TN + FP + FN).

In Table 2, results are shown for the mentioned metrics, where the results for all 50 test images, in original and reduced sizes, have been averaged. The last column shows the average processing time for each size. The DI is obtained as a value between 0 and 1, with 1 being the most accurate detection. It was transformed into percentage values to facilitate the comparison with the other metrics.

**Figure 7:**
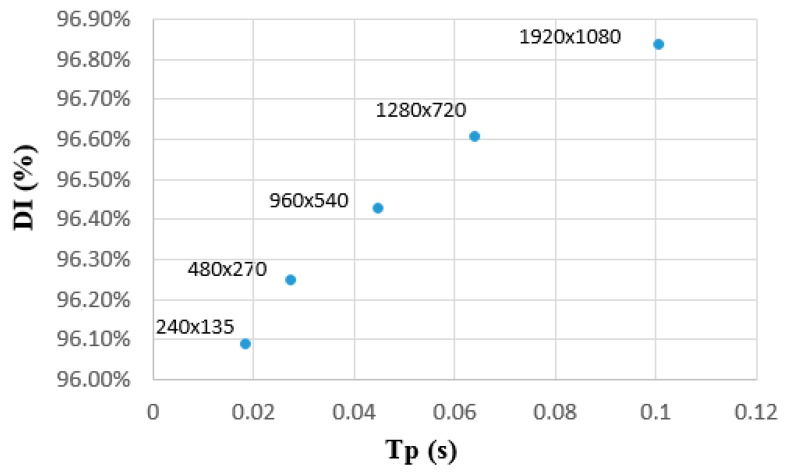
Relation between the general performance of the detection through Dice Index (DI) *vs.* time processing (Tp) for FFDI performance at different image resolutions.

For all image sizes, very high and similar rates are obtained for all metrics. It can be seen that the area detection accuracy diminishes as image size decreases, although it always maintains rates above 95% for *r*, *p,* and DI. For all sizes, JI is higher than 92% while the overall rate achieved with MI is between 97% and 98%. Nevertheless, a much faster detection can be accomplished with small sizes, reducing calculation time up to a fifth compared to the original image size. Consequently, with very small images, extremely high processing rates could be achieved and up to 54 images per second could be processed, which is significantly greater than the rate required for real-time, considering a general rate of 30 f/s for smooth video. In fact, 30 f/s would correspond to an approximate image size of 600 × 300 pixels, which is perfectly representative of a mobile device. Figure 7 shows graphically the relation between the values obtained for DI and processing time.

The presence of FP (false positives) has been found to occur principally in areas close to the forest fire. The explanation for this behavior is that the environment of the flame contains higher values in the red component due to reflections, such that confusion occurs at the time of detection. Other factors influencing the presence of FP may include changes in lighting and RGB chromaticity. Furthermore, an inexact calibration of the parameter *ρ* could contribute to the loss of information.

### 4.3. Detection of Early Stage Forest Fires

The way in which a fire spreads (speed, direction, *etc.*) and the distribution of its smoke, strongly depend on weather conditions which consequently influence the detection quality and rate. For instance, a smoke column observed from a distance could indicate an arousing fire, but in a windy environment, the smoke would spread rapidly. The flame, however, is a more secure source of information than smoke, as the wind rekindles it and its color is easier to detect, although it is not possible to observe it from a distance while the fire remains small. Here, the use of small UASs is a significant advantage, as they can fly at low altitudes and take images from inside views of the forest, through the trees.

Thirty test images were selected to test situations of early stages of fires. Table 3 shows numerical results for the same metrics calculated in the previous section. As real-time applications require fast processing times, only the two medium-sized images (960 × 540 and 480 × 270 pixels) were selected, due to their shorter processing times. For the same reasons as stated in Section 4.2, the evaluation has been performed with *ρ_min_*.

As can be seen, the results for these kinds of images show similar detection accuracies as for larger distance pictures. However, a reduction in the rate of FNs can be observed due to the improvement of Recall and JI, while maintaining similar processing times as those in Table 3 (the frame rate is slightly improved).

**Figure 8:**
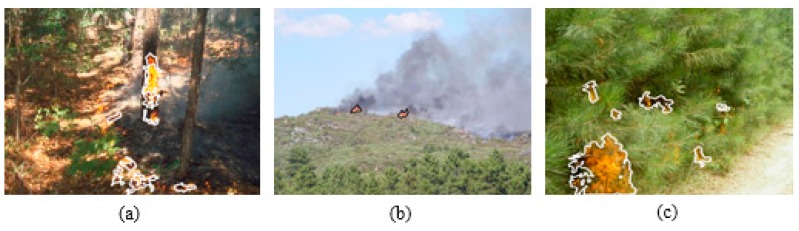
Detections of initial forest fires, realized with FFDI at short distances with; (**a**) *ρ_min_* = 0.2; (**b**) *ρ_min_* = 0.7; (**c**) *ρ_min_* = 1.

Figure 8 shows some visual examples extracted from the test set. The figure on the left has been obtained with *ρ_min_* = 0.2, which permits the detection of an initiating fire. The flame is not confused with the reddish tonalities of the ground using this *ρ*. Nevertheless, higher values of *ρ* are producing FPs.

The central image presents even smaller areas of flames, but they are clearly distinguishable from the green, gray and blue tonalities which dominate the histogram. In this case, *ρ_min_* = 0.7, higher values are resulting in FP due to confusion. Finally, the right image shows an example for a bush-fire, whose flames are directly distinguishable against the green background. The white road is not detected using *ρ_min_* = 1, as the color components are nearly equal (red mean = 234.17, green mean = 234.13 and blue mean = 211 are the average values of the histogram of this area).

The variation of *ρ* in the different images is because they have been obtained with different sensors, different resolutions, calibrations, sizes, *etc.* The environmental conditions, for instance, lighting, time of day and so forth also differed. It has been proven that *ρ_min_* and *ρ_max_* remain constant in image sequences obtained with the same sensor and under the same illumination conditions. It is also important to highlight that the *ρ_min_* should be fixed for applications in real-time.

### 4.4. Application to Non-Forested Environments

In Section 2, the development of both indices, FDI and FFDI has been explained, also indicating the insufficiency of FDI for precise detections in forested environments. Although the primary focus of this paper is on forest fire detection, some additional test results were also included for other environments, using images provided by the Data Bases and Images Group of the University of São Paulo (São Paulo, Brazil) [33].

**Figure 9:**
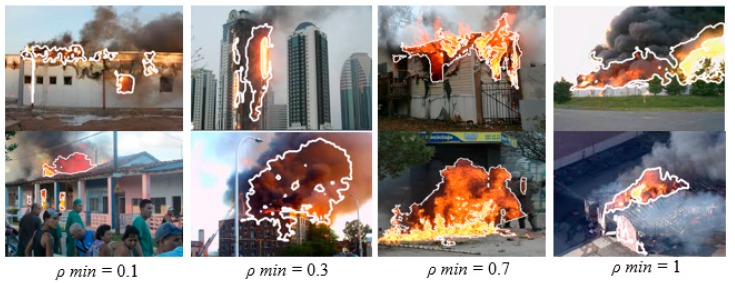
Fire detection with FFDI in different scenes.

The key to successful fire detection in other scenarios lies in Equation (6). As previously explained, values of *ρ* ≤ 1 assure flame detection, due to an enhancement of reddish and yellow colors, independently of the environment colors. In this way, the method also works for urbanized scenes with edifications and streets, as shown in Figure 9. Nevertheless, those are still preliminary tests, and they should be improved and adapted to the characteristics of these environments to achieve a reliable detection.

## 5. Discussion

Evaluation of the proposed method is undertaken in four stages: first analyzing the factor *ρ,* which aims to adjust the FFDI to different conditions, concretely to detect more or less of the smoke surrounding the flame.

The second and basic analysis used to show the applicability of the method in UASs resulted in very promising detection rates and processing times for aerial images displaying forest fires. It is evident that the method is valid for real-time use in UASs, due to achieving detection rates around 97% for frame rates up to 30 images per second. This means that information could be evaluated instantly, and alarms can be sent immediately from any location. The processing method of evaluating regions could also provide additional information such as the size of the burning zone or direction of propagation when a sequence of images is compared. In image sequences, different motion estimation techniques could be applied, as e.g., optical flow or block matching. Optical flow methods [34] are very precise as they calculate the gradients (motion direction) of all pixels, but carry the disadvantage to be highly cost intensive such that the advantage of real-time performance would fail. Probably, a fast block matching algorithm would be a better solution as the aim is to get a rough direction of the fire’s expansion and its velocity. For this purpose, a drone has a clear advantage over a plane, as it can stand still over a certain point during a desired amount of time.

Regarding the detection of already burned areas, a new index would be created, therefore, similar as explained in Section 2.2.1, Section 2.2.2, Section 2.2.3 and Section 2.2.4, images of destroyed forest would be analyzed to work out a new index for burned areas. Probably it would not be possible to adapt the FFDI and detect fire, smoke and burned areas in one step, but the objectives are also different, the FFDI aims at detecting and surveilling fires (application before and during a fire-event), a new index for burned areas would aim at exploring the regions after the event.

The third part of evaluation has focused on closer views corresponding to low altitude flights over areas with difficult access, which is possible with UASs/UAVs but not with conventional surveillance systems as manned planes or satellites [9,16,17]. Here, very high detection rates at the same low processing times have also been obtained.

Lastly to show its versatility, the method has also been evaluated successfully on images showing environments other than forest, such as edifications.

Summarizing, the proposed method seems quite promising for applications in UASs, as, besides efficiency, there are various additional benefits, such as the possibility of reducing costs of expensive equipment and manned flights, in turn reducing risks to personnel. According to a cost-benefit analysis performed by [19], the use of UAVs/UASs would save 33% of the costs incurred by employing helicopters.

## 6. Conclusions and Future Work

This study aims at providing an efficient and reliable method for forest fire detection that could be implemented in UASs and used in real-time. Therefore, two detection indices have been developed: the FDI (Fire Detection Index) and an extension of it, the FFDI (Forest Fire Detection Index), specially developed for vegetative environments. The authors propose the use of the FFDI for forest fire detection, as it shows very promising results and proves the possibility of real-time application, achieving overall detection rates of DI = 96.84% (1920 × 1080) at a maximum frame rate of 54 f/s for tiny image sizes (240 × 135).

A special feature that shows the FFDI is the use of the variable factor *ρ*, which permits an adaptive detection of the flame and the surrounding smoke. Depending on the condition and purpose of the application, this factor can be adjusted as required. In this way, FFDI can also be applied to environments other than forests, such as urbanizations.

Multiple evaluative tests performed over imagery obtained in different environments show the flexibility and adaptability of the method to a multitude of applications. Implemented in UASs, the algorithm could be employed for urbanized areas as well as for forests, and this has been proven to work well for distant and close views. The last point is especially interesting, as small UASs, also called drones, can easily access difficult or hidden places, where neither people nor vehicles nor planes could enter. Therefore, not only does the system outperform fixed, conventional systems placed on towers, as it is more flexible and covers a wider area, it is also much more cost-efficient than systems currently used in planes, helicopters or satellites: in other words, this algorithm achieves the same results more cheaply compared to existing systems.

These very promising results should encourage further development, with the method being implemented in a real-time system. Initial efforts employed a commercial drone type Bebop Parrot and a mobile control and evaluation platform (quad-core tablet with 2 GB memory and Android OS). The lightweight drone (420 g) is equipped with a high-resolution optical sensor with a frame rate of 30 f/s, 180° vision and video stabilizer, a wireless transmission system 802.11a/b/g/n/ac, geo-stabilization and geo-positioning system, vibration suppression, pre-configuration and flight autonomy.

The information flow in this system would be as follows: the UAS obtains the images and sends them via Wi-Fi to the mobile platform that runs the detection software and is also used as the control device for the drone. The video frames can be obtained at a maximum frequency of 30 f/s. The transmission rate of the wireless access point is 100 Mbps, such that the maximum image size at the greatest capture frequency would be 3.3 Mb, which allows much bigger images than the largest ones tested, which only need about 600 kb with jpeg compression. As the processing time of the detection algorithm reaches 22 f/s for 960 × 540 pixel sized images and 36 f/s for 480 × 270 pixels, an intermediate size will be proposed to get the maximum performance. As the channel allows much larger image formats, consequently, the challenge is to speed up the algorithm to obtain higher precision segmentations. It is also expected that there will be additional studies regarding a wider possible range of applications for FDI to scenarios other than forests.

A further challenge for future work is to obtain data about the burning and already burned area and provide it in real-time to the surveillance stations and fire workers, as it will be of great interest to estimate the dimension of a disaster in explorations after a fire. The geolocalization data is available through the GPS and the accelerometer mounted in the UAS. The area of the fire could be calculated through height information and image size/resolution, which leads to the relation of the pixel size to the corresponding area, such that the size of the detected RoI is equivalent to the size of the fire. An additional analysis of the pixel colors could also reveal facts about temperature or type of combustion, as different materials produce different flame colors in forested areas.

As already indicated in the discussion section, through video analysis it is possible to obtain the direction and the speed of the fire’s expansion. All in all, it is valuable information, which, could be obtained shortly in real-time.

## Figures and Tables

**Table 1:** Color component analysis for flame and smoke regions.

	*ρ*	Bright Yellow	Yellow	Orange	Orange Brown	Red Brown	Brown	Dark Grey	Brown Grey	Grey	Bright Grey	Blue Grey
R		244	244	252	205	189	114	134	154	186	232	199
G		221	203	170	93	34	67	115	137	178	211	207
B		157	71	46	47	21	51	92	111	159	204	245
r		0.39	0.47	0.54	0.59	0.77	0.49	0.39	0.38	0.36	0.36	0.31
g		0.36	0.39	0.36	0.27	0.14	0.29	0.34	0.34	0.34	0.33	0.32
b		0.25	0.14	0.10	0.14	0.09	0.22	0.27	0.28	0.30	0.32	0.38
ExG		0.07	0.18	0.09	−0.19	−0.58	−0.13	0.01	0.02	0.02	−0.02	−0.05
FDI		0.18	0.41	0.62	0.78	1.32	0.47	0.18	0.15	0.07	0.08	−0.08
FFDI	0	−0.07	−0.18	−0.09	0.19	0.58	0.13	−0.01	−0.02	−0.02	0.02	0.05
FFDI	0.5	0.02	0.03	0.22	0.58	1.24	0.37	0.08	0.05	0.01	0.06	0.00
FFDI	1	0.11	0.24	0.53	0.97	1.91	0.61	0.17	0.13	0.05	0.10	−0.04
FFDI	2	0.29	0.65	1.14	1.76	3.23	1.08	0.35	0.28	0.11	0.17	−0.12
**Binarization with TFFD = 0.158**												
BIN IMG	0	0	0	0	1	1	1	0	0	0	1	1
BIN IMG	0.5	1	1	1	1	1	1	1	1	1	1	1
BIN IMG	1	1	1	1	1	1	1	1	1	1	1	0
BIN IMG	2	1	1	1	1	1	1	1	1	1	1	0

**Table 2:** Average metrics obtained over 50 resized test images.

Size	r	*p*	DI	JI	MI	Tp (s)	f/s
1920 × 1080	96.30%	97.39%	96.84%	93.88%	97.67%	0.1005	10
1280 × 720	96.17%	97.05%	96.61%	93.44%	97.50%	0.0639	15
960 × 540	95.95%	96.82%	96.43%	93.02%	97.33%	0.0447	22
480 × 270	95.83%	96.70%	96.25%	92.80%	97.25%	0.0275	36
240 × 135	95.71%	96.47%	96.09%	92.48%	97.12%	0.0185	54

**Table 3:** Average metrics obtained over 30 images of initiating fire processes.

Size	r	*p*	DI	JI	Manhattan	Tp(s)	f/s
960 × 540	97.18%	96.62%	96.90%	93.98%	97.55%	0.0441	22
480 × 270	96.90%	96.62%	96.76%	93.73%	97.44%	0.0181	55

## References

[1] Lydersen J.M., North M.P., Collins B.M. (2014). Severity of an uncharacteristically large wildfire, the Rim Fire, in forests with relatively restored frequent fire regimes. For. Ecol. Manag..

[2] European Forest Fire Information System (EFFIS). http://forest.jrc.ec.europa.eu/effis/reports/.

[3] Flannigan M.D., Stocks B.J., Wotton B.M. (2000). Climate change and forest fires. Sci. Tot. Environ..

[4] Podur J., Wotton M. (2010). Will climate change overwhelm fire management capacity?. Ecol. Model..

[5] Collins B.M. (2014). Fire weather and large fire potential in the northern Sierra Nevada. Agric. For. Meteorol..

[6] Cappellini V., Mattii L., Mecocci A. (1989). An Intelligent System for Automatic Fire Detection in Forests. Recent Issues in Pattern Analysis and Recognition.

[7] Millan L., Sanchez G., Nakano M., Toscano-Medina K., Perez-Meana H., Rojas L. (2012). An early fire detection algorithm using IP cameras. Sensor.

[8] Sahin Y.G., Ince T. (2009). Early forest fire detection using radio-acoustic sounding system. Sensor.

[9] Yuan C., Zhang Y., Liu Z. (2015). A Survey on Technologies for Automatic Forest Fire Monitoring, Detection and Fighting Using UAVs and Remote Sensing Techniques. Can. J. For. Res..

[10] Qian Y., Yan G., Duan S., Kong X. (2009). A contextual fire detection algorithm for simulated HJ-1B imagery. Sensor.

[11] Yoon S.H. (2013). An intelligent automatic early detection system of forest fire smoke signatures using Gaussian mixture model. J. Inf. Process. Syst..

[12] Stula M., Krstinic D., Seric L. (2012). Intelligent forest fire monitoring system. Inf. Syst. Front..

[13] Arrue B.C., Ollero A., De Dios J. (2000). An intelligent system for false alarm reduction in infrared forest-fire detection. IEEE Int. Syst. Appl..

[14] Guijarro M., Pajares G., Riomoros I., Herrera P., Burgos-Artizzu X., Ribeiro A. (2011). Automatic segmentation of relevant textures in agricultural images. Comput. Elect. Agric..

[15] Montalvo M., Pajares G., Guerrero J.M., Romeo J., Guijarro M., Ribeiro A., Cruz J.M. (2012). Automatic detection of crop rows in maize fields with high weeds pressure. Exp. Syst. Appl..

[16] Tang L., Shao G. (2015). Drone remote sensing for forestry research and practices. J. For. Res..

[17] Salamí E., Barrado C., Pastor E. (2014). UAV flight experiments applied to the remote sensing of vegetated areas. Remote Sens..

[18] Watts A.C., Ambrosia V., Hinkley E.A. (2012). Unmanned aircraft systems in remote sensing and scientific research: Classification and considerations of use. Remote Sens..

[19] Christensen B.R. (2015). Use of UAV or remotely piloted aircraft and forward-looking infrared in forest, rural and wildland fire management: Evaluation using simple economic analysis. N. Z. J. For. Sci..

[20] Martínez-de Dios J.R., Merino L., Caballero F., Ollero A. (2011). Automatic forest-fire measuring using ground stations and unmanned aerial systems. Sensor.

[21] NASA Earth Science Division. http://geo.arc.nasa.gov/sge/UAVFiRE/completeddemos.html.

[22] Torres-Sánchez J., Peña J.M., de Castro A.I., López-Granados F. (2014). Multi-temporal mapping of the vegetation fraction in early season wheat fields using images from UAV. Comput. Elect. Agric..

[23] Peña J.M., Torres-Sánchez J., Serrano-Pérez A., de Castro A.I., López-Granados F. (2015). Quantifying efficacy and limits of unmanned aerial vehicle (UAV) technology for weed seedling detection as affected by sensor resolution. Sensor.

[24] Yuan C., Liu Z., Zhang Y. UAV-based forest fire detection and tracking using image processing techniques. Proceedings of the IEEE International Conference on Unmanned Aircraft Systems (ICUAS 2015).

[25] Woebbecke D., Meyer G., Von Bargen K., Mortensen D. (1995). Color indices for weed identification under various soil, residue, and lighting conditions. Trans. ASAE.

[26] Otsu N. (1979). A Threshold Selection Method from Gray-Level Histograms. IEEE Trans. Syst. Man Cyber..

[27] Meyer G.E., Hindman T.W., Laksmi K. (1999). Machine vision detection parameters for plant species identification. Proc. SPIE.

[28] Perez A.J., Lopez F., Benlloch J.V., Christensen S. (2000). Colour and shape analysis techniques for weed detection in cereal fields. Comput. Elect. Agric..

[29] Meyer G.E., Neto J.C. (2008). Verification of color vegetation indices for automated crop imaging applications. Comput. Elect. Agric..

[30] European Forest Fire Information System (EFFIS). http://forest.jrc.ec.europa.eu/effis/about-effis/technical-background/european-fire-database/.

[31] Warnell School of Forestry and Natural Resources, The University of Georgia, College of Agricultural and Environmental Sciences, Center for Invasive Species and Ecosystem Health, US Forest Service, International Society of Arboriculture, USDA Identification Technology Program Forestry Images Organization. http://www.forestryimages.org/browse/subimages.cfm?sub=740.

[32] Followgreenliving.com. http://followgreenliving.com/causes-forest-fire-nature-human-beings/.

[33] Database and Images Group ICMC/USP. http://www.gbdi.icmc.usp.br/downloads-en.html.

[34] Lucas B.D., Kanade T. An iterative image registration technique with an application to stereo vision. Proceedings of the International Joint Conference on Artificial Intelligence.

